# The Story of the Insane from Year to Year

**Published:** 1896-11-28

**Authors:** 


### THE STORY OF THE INSANE FROM YEAR TO YEAR.

NINTH SERIES.

### STAFFORD COUNTY ASYLUM AT LICHFIELD.

Here the numbers rose from 611 to 639. There were 200 admissions, 23 readmissions, 63 discharged recovered, and 121 deaths. The recoveries, calculated in the usual way, work out at 33·33 per cent., and the deaths at 19·57 per cent. The recovery rate is therefore below the average, and the death rate much above it. Of the deaths 67 were due to cerebral and spinal diseases, 21 to consumption, and 3 to pneumonia. As to the causes of insanity in the year's admissions we find that various moral causes account for 30; intemperance in drink for 33, and hereditary influence for 36. The asylum is full, and Dr. Spencer says the pressure for accommodation is becoming severely felt. New wards for female patients are being built, and when these are opened some relief will be experienced; but it is to be feared that the new asylum at Cheddleton was not begun nearly soon enough. The committee desire to record their high appreciation of the attention Dr. Spence continues to give to all the varied matters connected with the asylum. The Head Nurse retired on a pension of £30 a year. The weekly cost of maintenance is 9s. 0⅝d. per head.

### THE LANCASTER MOOR ASYLUM.

On January 1st the numbers were 1,809, and on December 31st these had risen to 1,877. There were 384 admissions, 54 readmissions, 114 recoveries, 52 relieved, and 156 deaths. The percentage of recoveries on admissions was 26·03, and the deaths stood at 8·94 on the average number resident. Of the deaths 49 were caused by cerebral and spinal diseases, 32 by consumption, 24 by pneumonia, and 16 by enteritis. Domestic troubles, adverse circumstances, love, &c., account for 90 of the admissions, intemperance in drink for 62, and hereditary influence in 100. That is in nearly one-half of the total admissions the causes were preventable. There is no reason why in a higher, better state of civilisation, both heredity and drink should not be struck off our asylum lists as producers of insanity; but we are ever chasing the shadow and neglecting the substance. Dr. Cassidy tells us of one of his patients who had five children, and three of these were epileptics, and the same patient had a brother who was in another asylum, while nothing is commoner in our county asylums than to find recurrent cases admitted every two or three years, who have had children in the meantime. The weekly cost per head is 8s. 7d.

THE ARGYLL AND BUTE ASYLUM.

There were 410 patients in residence on December 31st, 1895, being fewer by 3 than on January 1st. There were 62 admissions, 13 readmissions, 34 recoveries, 10 discharged relieved, and 31 deaths. The percentage of recoveries on admissions was 30, and that of the deaths on the average number resident was 12. Only 4 of the deaths were caused by cerebral diseases, and only 3 by consumption. Pneumonia was the cause in 4, intemperance in drink caused the insanity in 5 of the year's admissions, and hereditary influence in 10. Dr. Cameron continues the courses of instruction to the attendants and nurses, and he says "a marked improvement has taken place as regards the nursing of the patients, and a greater interest is evidently taken by the attendants and nurses." This has been noticed in other asylums, and it has been fostered in some by giving higher wages to those who have passed the examinations. The weekly maintenance rate is 7s. 8d. per head.

JOINT LUNATIC ASYLUM AT ABERGAVENNY.

An increase from 944 to 969 has to be noted during the year 1895. There were 201 admissions, but 40 of them were not first admissions; 77 recoveries, 8 discharged relieved, and 82 deaths. The recoveries stand at 38·3 per cent. on the admissions, and the deaths are 8·5 on the average number resident. Thirty of the deaths were from cerebral and spinal diseases, 6 from phthisis, and 16 from other lung diseases. Hereditary influence was the cause of the insanity in 39 of the admissions, drink in 22, and domestic trouble in 9. The partnership between the three counties has been dissolved. Monmouth will stand alone, and Brecon and Radnor will have to build. As the Monmouth patients number 675, the asylum will not be a small one, and with 60 private patients it will contain quite enough to be under one roof. Indeed, with the usual annual increment it will not take very many years to bring it to a thousand again. The asylum would seem to be in a very efficient state, and the committee "express their appreciation of the services of the Medical Superintendent." The weekly cost per head is 7s. 4¾d.

SALOP AND MONTGOMERY ASYLUM.

On January 1st, 1895, this asylum contained 784 patients, and on December 31st there were 810 in residence. There were 146 admissions, 51 readmissions, 35 transfers, 65 recoveries, 21 relieved, and 94 deaths. The recoveries were 33 per cent. on the admissions, and the deaths were 12·11 on the average number resident, showing that the recoveries were below the average and the deaths above it. Hereditary influence alone, or combined with other causes, accounts for 38 of the year's admissions, drink for 27, and 73 are put down as of unknown cause; consequently the table is not of much use. Of the deaths, 27 were caused by brain diseases, 8 by pneumonia, and 9 by phthisis. Dr. Strange has had a somewhat eventful year. A fire broke out in the laundry, causing damage to the extent of £500; influenza appeared again, and 70 of the patients and staff were affected in a few weeks; the storekeeper left for a better appointment in the Wilts County Asylum; and, what we do not remember to have noticed before in any asylum report, a female patient brought to the asylum was found to be dead on arrival. There is a profit on the farm of £957. This would be cheering news to the English farmer, but on looking over the accounts we see nothing charged for rent, nothing for interest on capital, and, except the gardener's wages, nothing for labour. On the other hand, bacon supplied to the house is put down at 6d. a pound and pork at 6½d. The visitors say they have again the pleasure of recording their appreciation of the manner in which the superintendent has performed his duties. The average cost per head per week is 7s. 11d. There are 23 private patients in the asylum, and these pay 15s. a week.

THE ROXBURGH DISTRICT ASYLUM.

This is a small asylum, containing at the close of the year only 253 patients. There were 28 admissions, 15 recoveries, and 15 deaths. The recoveries stand at 47·3 per cent. on the admissions, and the deaths at 6 per cent. on the average number resident. Cerebral and spinal diseases account for 8 of the deaths, and consumption for 1. Of the admissions, 11 owed their insanity to moral causes; 2 to intemperance in drink, and 21 to heredity. Scarlet fever broke out in the asylum, and six of the patients and staff were affected. The origin of the outbreak could not be traced. The weekly cost of maintenance was 9s. 6d.

